# *Ficus deltoidea* extract down-regulates protein tyrosine phosphatase 1B expression in a rat model of type 2 diabetes mellitus: a new insight into its antidiabetic mechanism

**DOI:** 10.1017/jns.2019.40

**Published:** 2020-01-20

**Authors:** Rehab F. Abdel-Rahman, Shahira M. Ezzat, Hanan A. Ogaly, Reham M. Abd-Elsalam, Alyaa F. Hessin, Mostafa I. Fekry, Dina F. Mansour, Shanaz O. Mohamed

**Affiliations:** 1 Pharmacology Department, National Research Centre, Giza, Egypt; 2 Pharmacognosy Department, Faculty of Pharmacy, Cairo University, Kasr El-Einy Street, Cairo 11562, Egypt; 3 Pharmacognosy Department, Faculty of Pharmacy, October University for Modern Sciences and Arts, 6th October Campus, 12566, Egypt; 4 Chemistry Department, College of Science, King Khalid University, Abha, Saudi Arabia; 5 Biochemistry Department, Faculty of Veterinary Medicine, Cairo University, Giza, Egypt; 6 Pathology Department, Faculty of Veterinary Medicine, Cairo University, Giza, Egypt; 7 Microbiology and Immunology Department, College of Medicine, University of Illinois, Chicago, IL, USA; 8 School of Pharmaceutical Sciences, Universiti Sains Malaysia, Pulau Pinang, Malaysia

**Keywords:** Dihydroxyolean-12-en-23-oic acid, *Ficus deltoidea*, Protein tyrosine phosphatase 1B, Glucose transporter-2, Phosphoenolpyruvate carboxykinase, Glucose 6-phosphatase, CAT, catalase, FBG, fasting blood glucose, FD, *Ficus deltoidea* var. *deltoidea* Jack, G6Pase, glucose 6-phosphatase, GPx, glutathione peroxidase, GSH, reduced glutathione, MDA, malondialdehyde, MET, metformin, NA, nicotinamide, PTP, protein tyrosine phosphatase, *Slc2a2*, GLUT2 gene, PEPCK, phosphoenolpyruvate carboxykinase, SOD, superoxide dismutase, STZ, streptozotocin, T2DM, type 2 diabetes mellitus

## Abstract

*Ficus deltoidea* var. *deltoidea* Jack (FD) is a well-known plant used in Malay folklore medicine to lower blood glucose in diabetic patients. For further research of the antihyperglycemic mechanisms, the protein tyrosine phosphatase 1B (PTP1B)-inhibitory effect of FD was analysed both *in vitro* and *in vivo.* To optimise a method for FD extraction, water, 50, 70, 80, 90 and 95 % ethanol extracts were prepared and determined for their total phenolic and triterpene contents, and PTP1B-inhibition capacity. Among the tested extracts, 70 % ethanol FD extract showed a significant PTP1B inhibition (92·0 % inhibition at 200 µg/ml) and high phenolic and triterpene contents. A bioassay-guided fractionation of the 70 % ethanol extract led to the isolation of a new triterpene (3β,11β-dihydroxyolean-12-en-23-oic acid; F3) along with six known compounds. *In vivo*, 4 weeks’ administration of 70 % ethanol FD extract (125, 250 and 500 mg/kg/d) to streptozotocin–nicotinamide-induced type 2 diabetic rats reversed the abnormal changes of blood glucose, insulin, total Hb, GLUT2, lipid profile, and oxidative stress in liver and pancreas. Moreover, FD reduced the mRNA expression of the key gluconeogenic enzymes (phosphoenolpyruvate carboxykinase and glucose 6-phosphatase) and restored insulin receptor and GLUT2 encoding gene (*Slc2a2*) expression. In addition, FD significantly down-regulated the hepatic PTP1B gene expression. These results revealed that FD could potentially improve insulin sensitivity, suppress hepatic glucose output and enhance glucose uptake in type 2 diabetes mellitus through down-regulation of PTP1B. Together, our findings give scientific evidence for the traditional use of FD as an antidiabetic agent.

Diabetes is a widespread, chronic and possibly life-threatening endocrine disease if left untreated. Globally, its prevalence continues to increase due to ageing and socio-economic changes^(1)^. According to the International Diabetes Federation (2010–2012), more than 300 million people have diabetes, representing 6 % of the world's adult population, and the global incidence is rapidly increasing. An additional seven million people develop the disease each year. The International Diabetes Federation estimates that 380 million people will be diagnosed with diabetes by 2025, with the greatest burden in low- and middle-income countries. Diabetes causes devastating complications, including amputations, kidney disease and heart disease, which can cause premature death in both children and adults. The cost of diabetes is a challenge for healthcare systems, even in the wealthiest countries^(2)^.

Type 2 diabetes mellitus (T2DM) is a serious health threat, particularly in modern society, and it is associated with impaired glucose metabolism (hyperglycaemia). It causes many complications, including CVD, blindness, renal failure and peripheral nerve damage^(3)^. Accordingly, intensive research and drug intervention strategies have been applied to develop potentially effective treatments for T2DM^(4)^.

Insulin resistance is a characteristic feature in the pathogenesis of T2DM and is characterised by defects in the peripheral glucose utilisation and development of hyperglycaemia. Therefore, insulin sensitisers, such as thiazolidinediones (or glitazones) have been widely used for T2DM treatment^(5)^. Numerous factors have been reported to impair the insulin signalling pathway by inhibiting the activation, or by suppressing the expression of signalling molecules. A key negative regulator of insulin signalling is protein tyrosine phosphatase 1B (PTP1B) that causes dephosphorylation of activated insulin receptor and induction of insulin resistance. Based on the overwhelming evidence, PTP1B inhibitors are anticipated to become potential therapeutic agents to control T2DM^(6,7)^.

One of the most popular and well-known plants with a long history of use among the Malays is *Ficus deltoidea* var. *deltoidea* Jack (FD), a plant of the family Moraceae. FD has been used as a medicine for various ailments in the Malay archipelago as well as distributed and formulated as capsules, teas and tonics throughout Malaysia^(8)^.

FD has been used to relieve headache, fever and toothache. A decoction of the whole plant has been used as a herbal drink to strengthen the uterus after birth in women^(9,10)^. Accumulating data have reported the blood glucose-lowering effect of FD due to an insulin-mimetic or insulinotropic activity^(11,12)^. Moreover, FD was demonstrated to inhibit intestinal α-glycosidase activity and block hepatic glucose production^(13)^. However, until this moment, there has been no report on the effect of FD on PTP1B activity or expression as a target insulin receptor signalling cascade.

The present study aims to elucidate the other molecular mechanisms of FD and to determine the possible involvement of PTP1B modulation in its glucose-lowering action against T2DM. To establish a relationship between pharmacological effects and bioactive constituents, phytochemical screening of various FD leaf extracts was performed via a bio-guided fractionation of the active extract to re-isolate and characterise novel triterpenes from FD and to evaluate their PTP1B-inhibiting activity.

## Materials and methods

### Chemicals

Ptp1b (human, recombinant), *p*-nitrophenyl phosphate, EDTA, citric acid, dithiothreitol, gallic acid (≥98·0 %) and ursolic acid (≥98·0 %) were obtained from Sigma Aldrich. Materials for chromatographic studies included pre-coated silica plates (60 GF 254; 20 × 20 cm) (Fluka for TLC, Diaion HP 20; Sigma-Aldrich Co.). Silica gel H (Merck) and Lichroprep RP-18 silica gel (15–25 µm; Merck) were used for vacuum liquid chromatography, and silica gel 60 (70–230 mesh ASTM; Fluka) and Sephadex LH-20 (Sigma-Aldrich Co.) for column chromatography. The following solvent systems were used for developing the chromatograms. S1: n-hexane–ethyl acetate (9:1, v/v); S2: methylene chloride–methanol (9·5:0·5, v/v); S3: ethylacetate–methanol–water (10:1·6:1·2, by vol.). Spots were visualised by spraying with *p*-anisaldehyde–sulfuric acid or natural products–polyethyleneglycol reagent (NP/PEG). ^1^H-NMR and ^13^C-NMR spectra were recorded on a Bruker high-performance digital Fourier transform-NMR spectrophotometer operating at 400 (^1^H) and 100 (^13^C) MHz in DMSO-d6 as a solvent and chemical shift were given in δ (parts per million) relative to solvent as an internal standard. The ^1^H-NMR was run for 1 h and ^13^C-NMR was run for 6 h at room temperature. Mass spectra were measured on a MS QQQ mass spectrometer equipped with an electrospray ion source in negative ion mode.

### Plant material and preparation of the crude extracts

The leaves of FD were obtained from HCA Products Sdn Bhd in spring 2015. The plant was kindly identified by Forest Research Institute, Malaysia. A voucher specimen (7-08-2015) was kept in the herbarium of the Pharmacognosy Department, Faculty of Pharmacy, Cairo University, Cairo, Egypt.

The powdered air-dried leaves of FD were extracted using water, and 50, 70, 80, 90 and 95 % ethanol (50 g powder for each solvent). The liquid–material ratio was 60:1 in a three-stage procedure (each in a ratio of 20:1), each time continued for 30 min using an ultrasonic bath at 60°C^(14)^. The combined extracts for each solvent were concentrated under reduced pressure using a rotary evaporator at 40°C to yield solid residues weighing 8·55, 8·51, 7·12, 4·77, 4·70 and 4·52 g of water, and 50, 70, 80, 90 and 95 % ethanol extracts.

### Estimation of total phenolics

The total phenolic content in the prepared six crude extracts of FD was estimated using Folin–Ciocalteu reagent^(15)^. The total phenolic content of each extract was separately calculated using the standard curve and expressed as gallic acid equivalents in mg/g of the extracts.

### Estimation of total triterpenes

Colorimetric estimation of total triterpene content was done according to Hiai *et al*.^(16)^ using vanillin reagent. The concentration of triterpenes was calculated as ursolic acid equivalents in mg/g extract with reference to a pre-established standard calibration curve.

### Fractionation and purification of active compounds

An amount of 5000 g FD leaves was extracted in 70 % ethanol as described in the previous section. Then, the extract was vacuum filtered, concentrated using a rotatory evaporator and lyophilised to yield a brownish yellow powder. Approximately 400 g of the dry extract suspended in 600 ml distilled water was subjected to liquid–liquid partitioning with dichloromethane (4 ×  1 litre) then evaporated using a rotary evaporator at 40°C. A quantity of 300 g of the dry extract was fractionated in a Diaion HP 20 chromatography column (60 cm length × 5 cm diameter, 500 g) using water–methanol mixtures (100:0, 75:25, 50:50, 25:75, 0:100, v/v) for elution. Desired fractions were pooled and evaporated at 40°C to yield 139·7, 66·6, 11·1, 6·0 and 2·41 g, respectively.

About 100 g of the above dichloromethane extract were separated in a vacuum liquid chromatography column using silica gel H (5 cm length × 10 cm diameter, 200 g). Gradient elution was performed using *n*-hexane, *n*-hexane–dichloromethane mixtures, dichloromethane, dichloromethane–ethyl acetate mixtures, ethyl acetate, ethyl acetate–methanol mixture and methanol. The polarity was increased by 5 % every 200 ml till 100 % methanol. Fractions (200 ml, each) were collected and monitored by TLC; similar fractions were pooled together to yield three sub-fractions (A–B). The three sub-fractions A, B and C were separately re-chromatographed over silica columns using different ratios of ethyl acetate–*n-*hexane (9, 10 and 15 % ethyl acetate in *n*-hexane, respectively) to yield three pure compounds F1, F2 and F3.

Fraction 75 % methanol in water from Diaion (1·0 g) was separated over a Sephadex column (LH-20; 30 cm length × 3 cm diameter) using methanol as eluent to yield three sub-fractions (I-III). These fractions were further purified on a Sephadex column (LH-20; 15 cm length × 2 cm diameter) using methanol–water (1:1, v/v) to obtain three pure compounds F4, F5 and F6.

### Protein tyrosine phosphatase 1B-inhibition assay

*In vitro* PTP1B-inhibition activity was determined using *p*-nitrophenyl phosphate as substrate^(17)^. Briefly, 50 mm-sodium citrate (pH 6·0), 0·1 mm-EDTA, 1 mm-dithiothreitol, 2 mm-*p*-nitrophenyl phosphate, 0·1 µg PTP1B and varying concentration of inhibitors (extracts, fractions, isolates or ursolic acid) up to 200 µl, were incubated at 37°C for 30 min, then the reaction was terminated by adding 10 m-NaOH. The amount of *p*-nitrophenol was monitored at 405 nm. The results are means of three measurements.

### Animals

Adult male Wistar rats (150–175 g), 3 months old, were obtained from the Animal House Colony at the National Research Centre (NRC) Egypt. All animals were housed under constant temperature and a 12-h light–dark cycle. They were fed a standard chow diet (Al-Marwa for Animals Feed Manufacturing) containing 19·80 % protein, 39·25 % carbohydrate, 4·41 % fat and 13·25 % fibres. After 1 week of acclimatisation, rats were randomly allocated into six groups (*n* 7 each). Animal procedures were performed according to the protocol approved by the Institutional Animal Care and Use Committee at Cairo University (approval number: CU-II-F-27-18) and the NRC Medical Ethics Committee (approval number: MREC-17-081) and following the recommendations of the National Institutes of Health Guide for Care and Use of Laboratory Animals (publication no. 85-23, revised 1985).

The experimental endpoint was set when the scientific aims and objectives had been reached. During the experimental study, we ensured that pain and distress were minimised. At the end of the study, euthanasia of rats was done by means that induce rapid unconsciousness and death without pain or distress through an intraperitoneal overdose of pentobarbital sodium (200 mg/kg, intraperitoneally).

### Selection of *Ficus deltoidea* doses

A preliminary toxicity study was performed by giving a group of rats FD extract, orally at a dose of 5 g/kg. No lethality was recorded and so we examined the antidiabetic activity of FD at one-tenth the highest dose which was non-toxic nor lethal, 1/20 and 1/40 in the present study.

### Experimental design

T2DM was induced by two consecutive injections of nicotinamide (NA) and streptozotocin (STZ)^(18)^. NA was dissolved in normal saline. Rats were intraperitoneally injected with NA (110 mg/kg) 15 min prior to the intraperitoneal injection of a freshly prepared solution of STZ (45 mg/kg) in 0·1 m-citrate buffer (pH 4·5) in overnight fasted rats^(19)^. All rats were injected with STZ–NA, except negative control rats, which received only the vehicle, distilled water^(20)^. After 6 h of NA injection, rats were provided with free access to glucose solution (10 %, w/v) for the next 24 h. After 48 h of STZ administration, fasting blood glucose (FBG) level was measured according to Trinder^(21)^. Rats having FBG values >200 mg/dl (>11·1 mmol/l) were considered diabetic and were assigned for the screening and assays^(19)^. Diabetic animals were randomly allocated into six groups, of seven rats each. Treatment was carried out for 4 weeks as follows: the 1st and the 2nd groups received only the vehicle (distilled water) orally and served as the normal and diabetic control groups, respectively. The 3rd group was orally administered metformin (MET; 150 mg/kg per d) as a reference control group. The 4th, 5th and 6th groups received 70 % ethanol extract of FD (125, 250 and 500 mg/kg per d) orally. FBG was measured 14 and 28 d after medication.

At the end of the 28th day, blood samples were withdrawn from the retro-orbital venous plexus under light ether anaesthesia into two sampling tubes, one containing Na-EDTA at day 28 post-medication for the estimation of Hb^(22)^. The second blood sample was centrifuged at 3500 rpm for 15 min to separate sera for the estimation of insulin level^(23)^. Other biochemical parameters such as alanine transaminase and aspartate transaminase activities in serum were measured^(24)^. Serum levels of total bilirubin^(25)^, total protein^(26)^, TAG^(27)^, total cholesterol^(28)^, HDL-cholesterol^(29)^ and LDL-cholesterol^(30)^ were measured using commercially available kits (Quimica Clinica). Preparation of pancreatic homogenate was done according to Mansour *et al*.^(31)^. The activities of superoxide dismutase (SOD), glutathione peroxidase (GPx) and catalase (CAT) in hepatic and pancreatic tissues were estimated^(32–34)^, respectively. Reduced glutathione (GSH)^(35)^, lipid peroxidation products were estimated by determining malondialdehyde (MDA) content in the hepatic and pancreatic tissue^(36)^ and GLUT2 was determined using commercial diagnostic kits (Chema Diagnostica)^(37)^.

### Histopathological examination

The pancreatic tissues from the different groups were fixed in 10 % neutral buffered formalin and routinely processed for paraffin embedding to obtain 4 µm sections. The tissue sections were stained with haematoxylin and eosin stain^(38)^. The histopathological lesion scoring of the pancreas was performed^(39)^.

### Immunohistochemical analysis

The immunohistochemical analysis was done following the methods described by Abdel-Rahman *et al*.^(40)^. The pancreatic tissue sections were deparaffinised and rehydrated. The endogenous peroxidase activity was blocked by adding a few drops of hydrogen peroxide (Thermo Scientific). The antigenic retrieval process was performed by pre-treating tissue sections with 10 mm-citrate buffer (pH 6·0) for 10 min in a microwave oven. Sections were incubated overnight at 4°C in a humidified chamber with one of the following primary antibodies: mouse monoclonal anti-insulin clone E11D7 (05-1066; Millipore) at a dilution of 1:50, mouse monoclonal anti-glucagon antibody clone 13D11.33 (MABN238; Millipore) at a dilution of 1:8000 and rat monoclonal anti-somatostatin antibody clone YC7 (MAB354; Millipore) at a dilution of 1:100. The sections were rinsed with PBS then incubated with a sheep anti-mouse antibody (AQ300D; Millipore) and goat anti-rat antibody (AP136P; Millipore) for 10 min. Finally, sections were incubated with streptavidin peroxidase (Thermo Scientific). The slides were incubated with 3,3′-diaminobenzidine tetrahydrochloride as chromogen (DAB; Sigma) for 10 min. The slides were counterstained with haematoxylin and mounted. In each field, the immunopositive (dark brown) area was recorded. Percentage of positive stained area (%) was calculated as mean of ten fields/slide. The morphometric analysis of the pancreatic islet cells composition was performed according to methods described by Mu *et al*.^(41)^ to determine the percentage of insulin-positive β-cells to the total islet area, glucagon-positive α-cells to the total islet area and the somatostatin-positive δ-cells to the total islet area.

### Gene-expression analysis

Total RNA was isolated from rat livers using an Rneasy Mini Kit according to the manufacturer's protocol (Qiagen). First-strand cDNA synthesis from 1 µg total RNA was done employing a reverse transcriptase kit and oligo(dT) (Thermo Scientific). *PTP1B*, phosphoenolpyruvate carboxykinase (*PEPCK*), glucose 6-phosphatase (*G6Pase*), GLUT2 (*Slc2a2*) and insulin receptor (*INR*) target genes were amplified by quantitative real-time-PCR using specific primers (Supplementary Table S1). cDNA was added to a Quantifast SYBR Green qPCR Master Mix (Qiagen) containing 30 pg/ml of each primer. The thermal profile included forty cycles of denaturation at 95°C for 15 s, annealing at 60°C for 15 s and extension at 72°C for 45 s. During the first cycle, the 95°C step was extended to 1 min. The β-actin gene was amplified in the same reaction as a reference gene to normalise data. Relative gene expression was calculated using the Livak & Schmittgen method^(42)^.

### Statistical analysis

All results are expressed as means and standard deviations. Multiple group comparisons were performed by ANOVA followed by Tukey's multiple comparison *post hoc* test (two-sided) at *P* ≤ 0·05. GraphPad prism® software (version 6.00 for Windows) was used.

## Results

### Estimation of total phenolic and triterpene contents

The 70 % ethanol extract contained the highest triterpene concentrations (420·49 (sd 1·08) mg ursolic acid equivalents/g extract), and the highest phenolic concentrations (222·6 (sd 0·37) mg gallic acid equivalents/g extract) (Table 1).

**Table 1.** Total phenolic and total triterpene contents of *Ficus deltoidea* extracts (Mean values and standard deviations; three replicates)

	Total phenolics (mg GA equivalents/g)		Total triterpenes (mg UA equivalents/g)	
Extract	Mean	sd	Mean	sd
Water	118·19	1·06	147·56	2·11
50% Ethanol	111·72	0·98	319·30	1·76
70% Ethanol	222·59	0·37	420·49	1·08
80% Ethanol	145·03	2·01	303·0	0·52
90% Ethanol	162·85	1·28	360·91	2·16
95% Ethanol	149·51	0·86	343·70	0·71

GA, gallic acid; UA, ursolic acid.

### Identification of isolated compounds

**Fig. 1.**
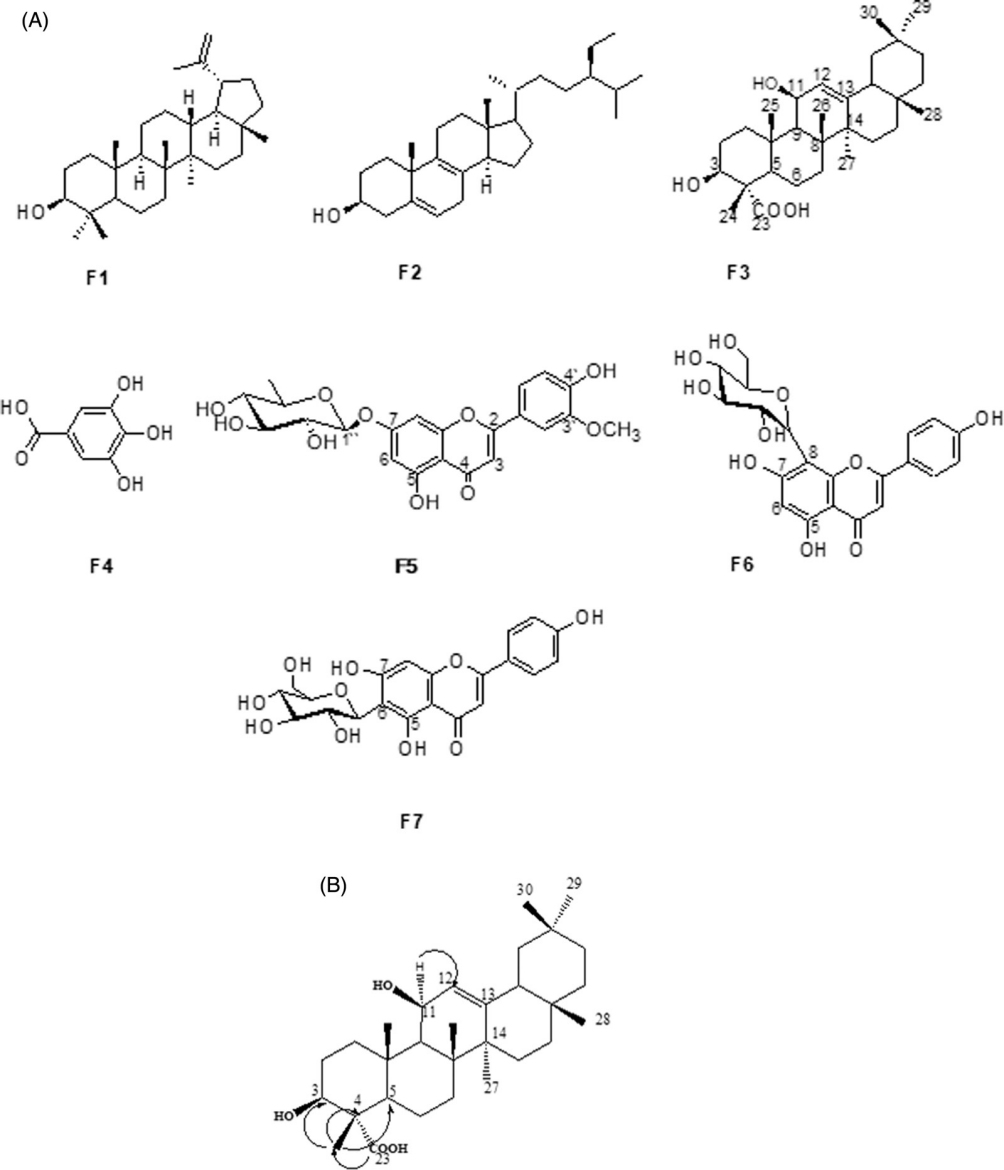
(A) Structures of the isolated compounds F1–F7. (B) Heteronuclear multiple bond correlations for F3.

Three compounds were isolated from the methylene chloride fraction: lupeol (F1), (24E)-stigmasta-5,8-dien-3β-ol (F2) and 3β,11β-dihydroxyolean-12-en-23-oic acid (F3) (Supplementary Table S2). Four compounds were isolated from the 75 % methanol fraction and were identified as gallic acid (F4), chryseriol-7O-α-rhamnoside (F5), vitexin (apigenin-8C-β-d-glucoside) (F6) and isovitexin (apigenin-6C-β-d-glucoside) (F7) (Supplementary Table S3). The isolated compounds were identified by analysing the spectroscopic data from one- and two-dimensional NMR, and MS experiments (Supplementary Table S4). The structures of the isolated compounds are presented in Fig. 1(A). The HMBC (heteronuclear multiple bond correlations) for F3 are shown in Fig. 1(B).

### Protein tyrosine phosphatase 1B-inhibitory effects of extracts and fractions

**Fig. 2.**
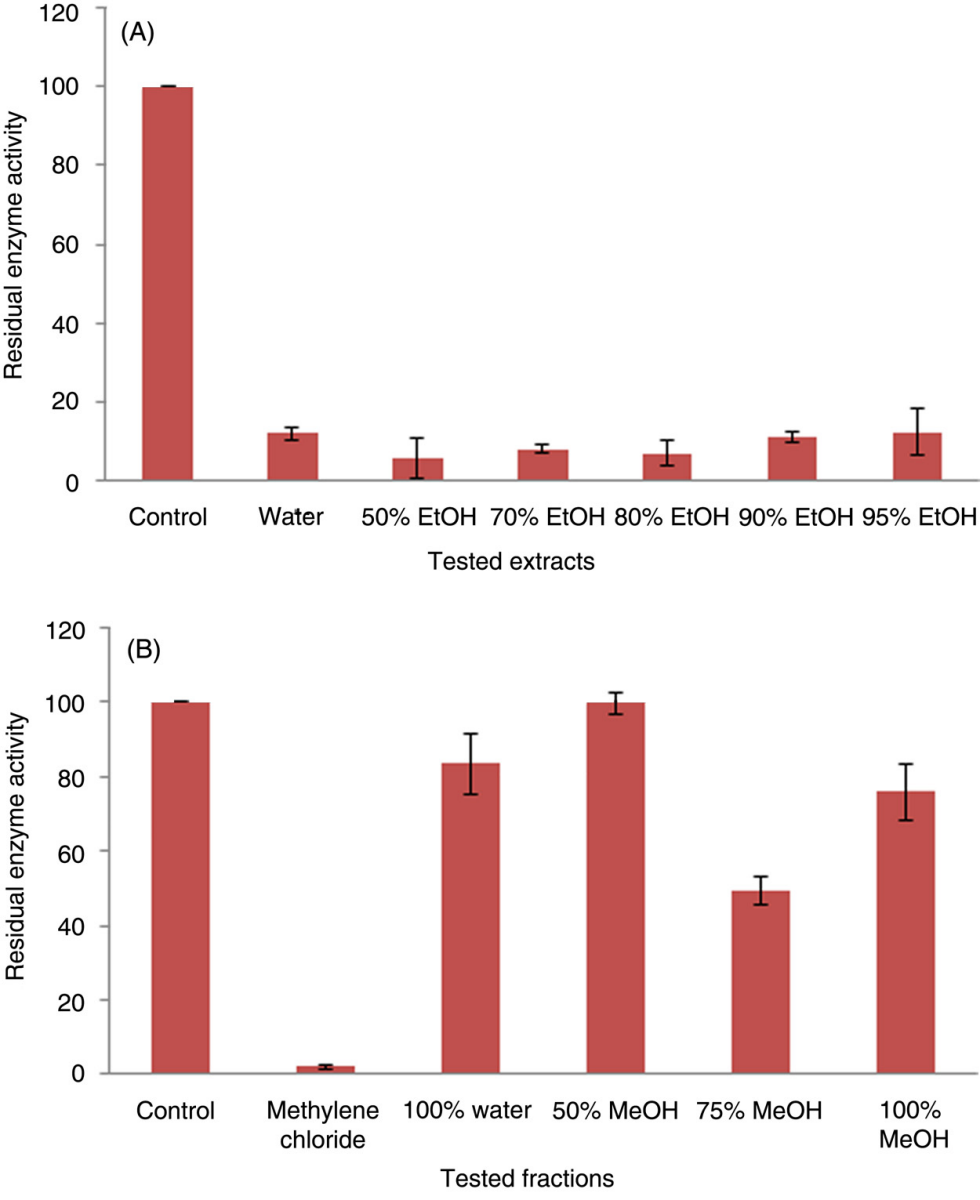
Protein tyrosine phosphatase 1B-inhibitory effects of extracts (A) and fractions (B). EtOH, ethanol; MeOH, methanol.

As presented in Fig. 2(A), high *in vitro* PTP1B inhibition (93·15, 92·0, and 94·36 %) was reported for 80, 70 and 50 % ethanol extracts, respectively. However, the aqueous, 90 and 95 % ethanol extracts showed relatively lower PTP1B-inhibition activities, with 88·06, 89·11 and 87·73 % inhibition, respectively.

The methylene chloride subfractions of the 70 % ethanol FD extracts showed the highest *in vitro* PTP1B-inhibition activity (97·88% inhibition), followed by 75 % methanol in water Diaion fraction (50·48% inhibition) (Fig. 2(B)). It is worthy to note that higher concentration could not be tested due to the interference of the extract colour with the coloured product of the PTP1B assay.

As shown in Table 2, compounds F1, F2, F3 and F7 inhibited PTP1B activity in a dose-dependent manner, with half maximal inhibitory concentration (IC_50_) values ranging from 2·88 (sd 0·16) to 83·67 (sd 14·85) µm. Moreover, the two new pentacyclic triterpenes, F3 and F1, inhibited PTP1B activity (IC_50_ 4·55 (sd 1·01) and 2·88 (sd 0·16) µm, respectively) to a similar extent as the standard ursolic acid (IC_50_ 3·64 (sd 0·53) µm) due to their similarities in structure, which certainly affects the binding to the active sites of the enzyme. A new triterpene, 3β,11β-dihydroxyolean-12-en-23-oic acid (F3), was isolated which may be a potent PTP1B inhibitor.

**Table 2.** Protein tyrosine phosphatase 1B-inhibitory activities of the *Ficus deltoidea* isolated compounds (Mean values and standard deviations)

	IC_50_ values (μm)	
Compound	Mean	sd
F1	2·88	0·16
F2	73·68	7·51
F3	4·55	1·01
F4	–	
F5	–	
F6	82·57	12·48
F7	83·67	14·85
Ursolic acid	3·64	0·53

IC_50_, half maximal inhibitory concentration.

### Effect of *Ficus deltoidea* on fasting blood glucose levels

FBG levels were significantly increased by 171 % after 48 h of STZ administration; this level was persistently and significantly increased for 4 weeks (the end of the experiment) compared with the normal blood glucose level. Treatment of diabetic rats with 70 % ethanol extract of FD at doses of 125, 250 and 500 mg/kg significantly reduced FBG levels compared with the diabetic control. Animals that had been treated with the antidiabetic drug, MET, for 4 weeks displayed normal FBG levels. Notably, FD lowered FBG levels to normal levels after 2 weeks of treatment, indicating a significant difference from MET (Table 3).

**Table 3.** Effect of *Ficus deltoidea* (FD) on fasting blood glucose (FBG), insulin and Hb (Mean values and standard deviations; *n* 7)

	FBG (mg/dl)§									
	Baseline		2 weeks		4 weeks		Insulin (mIU/ml)		Hb (g/l)	
Groups‡	Mean	sd	Mean	sd	Mean	sd	Mean	sd	Mean	sd
Normal control	96·5	6·72	93·0	11·42	79·5	10·78	18·9	4·12	142	10·1
Diabetic control	262·1*	52·34	203·0*	9·70	208·9*	5·08	6·3*	1·55	105*	8·8
MET	254·7*	60·96	164·7*†	41·16	82·0†	6·78	9·4*	1·78	135†	8·5
FD, 125 mg/kg	286·8*	32·99	73·8†	21·82	79·5†	11·00	13·1*†	1·69	133†	24·6
FD, 250 mg/kg	268·2*	34·21	80·5†	22·08	76·0†	8·07	12·0*†	1·93	141†	12·7
FD, 500 mg/kg	285·8*	35·35	72·0†	20·22	67·0†	11·97	10·7*†	1·76	147†	11·9

MET, metformin.

* Mean value was statistically significantly different from that of the normal control group at the corresponding time (*P* ≤ 0·05).

† Mean value was statistically significantly different from that of the diabetic control group at the corresponding time (*P* ≤ 0·05).

‡ Adult male Wistar rats received either distilled water (normal control) or streptozotocin (45 mg/kg) in citrate buffer (diabetic control) by intraperitoneal injection. Diabetic rats received FD (125, 250 or 500 mg/kg, orally), MET (150 mg/kg, orally) for 4 weeks, 48 h after induction of diabetes.

§ To convert glucose in mg/dl to mmol/l, multiply by 0·0555.

### Effect of *Ficus deltoidea* on insulin and total Hb levels

Diabetic control rats exhibited significant decreases in blood insulin and total Hb level to 66 and 28 % compared with the normal control. All doses of FD significantly increased plasma insulin levels compared with the diabetic control. Hb reached normal levels after 4 weeks of FD treatment. MET significantly increased insulin and Hb levels (Table 3).

### Effect of *Ficus deltoidea* on hepatic markers

Diabetic rats showed significant increases in the levels of enzyme markers of liver function and total bilirubin by 3- and 2-fold, respectively, whereas total protein content was decreased by 50 % in the diabetic control compared with the normal control group. Treatment for 4 weeks with 125, 250 and 500 mg/kg FD significantly reduced the levels of liver enzymes in a dose-dependent manner and normalised total bilirubin levels. A non-significant change in total protein content was recorded in FD-treated rats compared with the diabetic control. MET administration normalised the levels of alanine transaminase, aspartate transaminase and total bilirubin, with a significant elevation in total protein content compared with diabetic rats (Table 4).

**Table 4.** Effect of *Ficus deltoidea* (FD) on serum hepatic markers, bilirubin and total protein (Mean values and standard deviations; *n* 7)

	ALT (U/l)		AST (U/l)		Total bilirubin (mg/l)		Total protein (g/l)	
Groups‡	Mean	sd	Mean	sd	Mean	sd	Mean	sd
Normal control	37·6	4·58	75·9	10·71	6	2·0	166	26·4
Diabetic control	126·4*	12·37	205·6*	15·91	12*	2·5	84*	5·60
MET, 150 mg/kg	42·7†	4·38	69·5†	3·62	4†	1·2	114*	15·2
FD, 125 mg/kg	58·4*†	7·61	106·6*†	6·09	7†	0·7	86*	3·0
FD, 250 mg/kg	58·1*†	3·97	111·3†	11·27	5†	1·6	81*	8·2
FD, 500 mg/kg	48·4*†	3·25	90·6†	2·57	6†	3·0	88*	2·9

ALT, alanine transaminase; AST, aspartate transaminase; MET, metformin.

* Mean value was statistically significantly different from that of the normal control group (*P* ≤ 0·05).

† Mean value was statistically significantly different from that of the diabetic control group (*P* ≤ 0·05).

‡ Adult male Wistar rats received either distilled water (normal control) or streptozotocin (45 mg/kg) in citrate buffer (diabetic control) by intraperitoneal injection. Diabetic rats received FD (125, 250 or 500 mg/kg, orally), MET (150 mg/kg, orally) for 4 weeks, 48 h after induction of diabetes.

### Effect of *Ficus deltoidea* on lipid profile

Diabetic control rats showed significant increases in total cholesterol, TAG and LDL levels by 41, 71 and 121 %, respectively, but a significant reduction in HDL levels of 40 % compared with normal rats. FD significantly reduced total cholesterol and LDL levels and produced non-significant changes in TAG levels compared with the diabetic control. Normal HDL levels were recorded after the FD treatment. The MET-treated group displayed significantly reductions in lipid profile parameters to normal levels after 4 weeks of treatment compared with the diabetic control (Table 5).

**Table 5.** Effect of *Ficus deltoidea* (FD) on serum lipid profile (Mean values and standard deviations; *n* 7)

	Total cholesterol (mg/dl)§		TAG (mg/dl)§		HDL-cholesterol (mg/dl)§		LDL-cholesterol (mg/dl)§	
Groups‡	Mean	sd	Mean	sd	Mean	sd	Mean	sd
Normal control	62·9	6·34	51·0	8·96	28·5	3·80	19·3	2·40
Diabetic control	89·5*	6·15	87·5*	5·79	17·4*	3·3	42·2*	7·89
MET, 150 mg/kg	59·4†	5·19	57·8†	10·47	29·4†	2·66	20·8†	2·87
FD, 125 mg/kg	74·3*†	5·26	82·1*	10·30	25·0†	2·36	32·3*†	4·81
FD, 250 mg/kg	72·6†	9·45	86·3*	4·64	25·5†	4·70	32·5*†	1·75
FD, 500 mg/kg	69·9†	7·50	87·1*	6·28	30·4†	2·57	32·3*†	1·94

MET, metformin.

* Mean value was statistically significantly different from that of the normal control group (*P* ≤ 0·05).

† Mean value was statistically significantly different from that of the diabetic control group (*P* ≤ 0·05).

‡ Adult male Wistar rats received either distilled water (normal control) or streptozotocin (45 mg/kg) in citrate buffer (diabetic control) by intraperitoneal injection. Diabetic rats received FD (125, 250 or 500 mg/kg, orally), MET (150 mg/kg, orally) for 4 weeks, 48 h after induction of diabetes.

§ To convert cholesterol in mg/dl to mmol/l, multiply by 0·0259. To convert TAG in mg/dl to mmol/l, multiply by 0·0113.

### Effect of *Ficus deltoidea* on hepatic oxidative stress biomarkers

Oxidative stress was confirmed in diabetic rats after STZ administration as a significant decrease in GSH levels and increase in MDA levels compared with normal rats. Besides, STZ–NA diabetic rats displayed significantly decreased levels of the antioxidant enzymes CAT, SOD and GPx by 70, 66 and 74 %, respectively, compared with normal rats. Oral administration of 125, 250 or 500 mg/kg FD dose-dependently and significantly elevated the GSH level compared with diabetic rats. The FD treatment did not significantly affect the MDA level, with the exception that 250 mg/kg FD significantly reduced the hepatic MDA level in diabetic rats. There were significant elevations in CAT, SOD and GPx levels in all FD-treated groups compared with diabetic rats. MET increased the GSH content and antioxidant enzyme activities and decreased the MDA level compared with the diabetic control group (Table 6).

**Table 6.** Effect of *Ficus deltoidea* (FD) on hepatic oxidative stress biomarkers (Mean values and standard deviations; *n* 7)

	GSH (μg/g tissue)		MDA (nmol/g tissue)		CAT (U/g tissue)		SOD (U/g tissue)		GPx (mmol/g tissue)	
Groups‡	Mean	sd	Mean	sd	Mean	sd	Mean	sd	Mean	sd
Normal control	249·9	23·48	105·4	8·08	6·29	0·47	5·59	0·81	4·64	0·72
Diabetic control	135·2*	5·80	187·3*	7·47	1·85*	0·28	1·76*	0·26	1·25*	0·20
MET, 150 mg/kg	201·9*†	34·67	158·9*†	6·71	4·33*†	0·39	4·21*†	0·36	3·08*†	0·09
FD, 125 mg/kg	173·7*†	20·24	185·9*	11·76	5·53†	0·43	5·43†	0·37	3·53*†	0·49
FD, 250 mg/kg	183·3*†	9·8	164·6*†	8·47	4·96*†	0·60	3·63*†	0·47	3·91*†	0·23
FD, 500 mg/kg	208·8*†	31·63	169·5*	0·53	5·44*†	0·19	4·91†	0·22	3·93†	0·44

GSH, reduced glutathione; MDA, malondialdehyde; CAT, catalase; SOD, superoxide dismutase; GPx, glutathione peroxidase; MET, metformin.

* Mean value was statistically significantly different from that of the normal control group (*P* ≤ 0·05).

† Mean value was statistically significantly different from that of the diabetic control group (*P* ≤ 0·05).

‡ Adult male Wistar rats received either distilled water (normal control) or streptozotocin (45 mg/kg) in citrate buffer (diabetic control) by intraperitoneal injection. Diabetic rats received FD (125, 250 or 500 mg/kg, orally), MET (150 mg/kg, orally) for 4 weeks, 48 h after induction of diabetes.

### Effect of *Ficus deltoidea* on GLUT2 protein levels

**Fig. 3.**
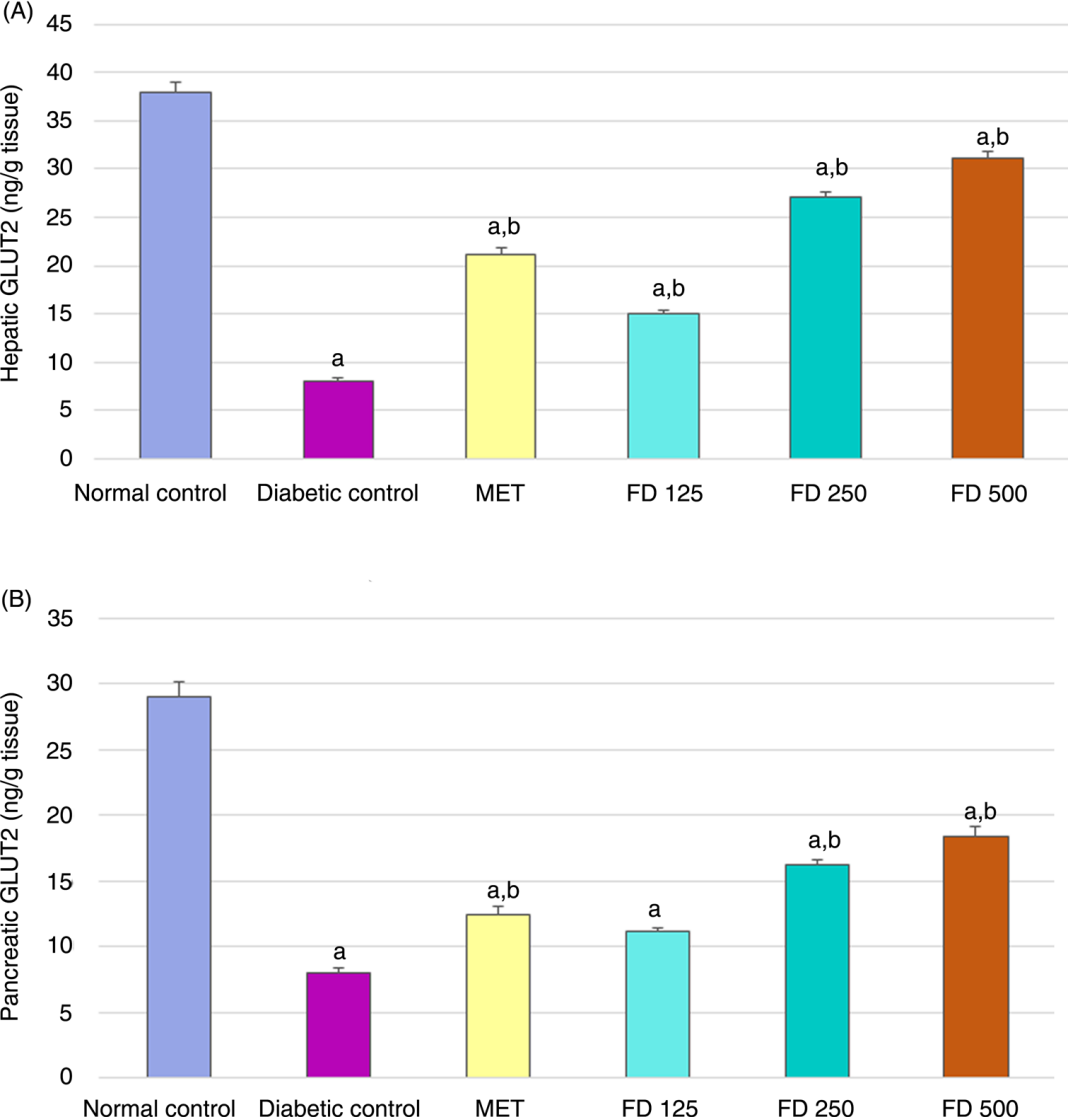
Hepatic (A) and pancreatic (B) GLUT2 concentrations. Values are means, with standard deviations represented by vertical bars. ^a,b^ Mean values with unlike letters were significantly different (*P* ≤0.05). MET, metformin; FD 125, *Ficus deltoidei* 125 mg/kg body weight; FD 250, *F. deltoidei* 250 mg/kg body weight; FD 500, *F. deltoidei* 500 mg/kg body weight.

Diabetic rats exhibited a significant, 80 and 73 % reduction in GLUT2 levels in the liver and pancreas compared with normal rats. Significant elevations in hepatic GLUT2 levels of 87, 237 and 287 % were recorded in diabetic rats treated with 125, 250 or 500 mg/kg FD, respectively. Treatment with 250 and 500 mg/kg FD led to significantly elevated pancreatic GLUT2 levels by 102 and 130 %, respectively, whereas 125 mg/kg FD had a non-significant effect on pancreatic GLUT2 levels. Furthermore, MET significantly increased GLUT2 in both the liver and pancreas, compared with the diabetic control (Figs 3(A) and (B)).

### Effect of *Ficus deltoidea* on pancreatic oxidative stress and antioxidant enzymes

After STZ administration, pancreatic tissues showed a significant, 38 % decrease in GSH levels and a 296 % increase in MDA levels with significant decreases in the levels of the antioxidant enzymes CAT, SOD and GPx of 65, 63 and 64 %, respectively, compared with normal rats. Diabetic rats orally treated with 125, 250 or 500 mg/kg FD exhibited dose-dependent increases in GSH levels of 39, 40 and 49 %, and significant increases in CAT levels of 60, 68 and 63 %, respectively, compared with diabetic rats. Moreover, 500 mg/kg FD significantly increased SOD activity by 54 % compared with diabetic rats, whereas 250 and 500 mg/kg FD significantly increased GPx levels by 66 and 75 %, respectively. In contrast, 150 mg/kg MET significantly increased GPx by 63 % compared with diabetic control rats (Table 7).

**Table 7.** Effect of *Ficus deltoidea* (FD) on pancreatic oxidative stress biomarkers (Mean values and standard deviations; *n* 7)

	GSH (μg/g tissue)		MDA (nmol/g tissue)		CAT (U/g tissue)		SOD (U/g tissue)		GPx (mmol/g tissue)	
Groups‡	Mean	sd	Mean	sd	Mean	sd	Mean	sd	Mean	sd
Normal control	192·4	13·60	24·0	3·65	5·34	0·61	4·76	0·67	3·88	0·17
Diabetic control	118*	2·04	95·6*	8·74	1·86*	0·46	1·74*	0·42	1·38*	0·31
MET, 150 mg/kg	171·9*†	14·43	86·2*	10·19	2·48*	0·34	2·33*	0·48	2·25*†	0·42
FD, 125 mg/kg	163·4*†	12·14	92·6*	7·26	2·99*†	0·46	2·19*	0·41	1·39*	0·26
FD, 250 mg/kg	166*†	10·46	81·3†	6·73	3·13*†	0·49	2·41*	0·54	2·29*†	0·32
FD, 500 mg/kg	176·1†	11·20	66·1*†	7·10	3·04*†	0·34	2·68*†	0·24	2·41*†	0·26

GSH, reduced glutathione; MDA, malondialdehyde; CAT, catalase; SOD, superoxide dismutase; GPx, glutathione peroxidase; MET, metformin.

* Mean value was statistically significantly different from that of the normal control group (*P* ≤ 0·05).

† Mean value was statistically significantly different from that of the diabetic control group (*P* ≤ 0·05).

‡ Adult male Wistar rats received either distilled water (normal control) or streptozotocin (45 mg/kg) in citrate buffer (diabetic control) by intraperitoneal injection. Diabetic rats received FD (125, 250 or 500 mg/kg, orally), MET (150 mg/kg, orally) for 4 weeks, 48 h after induction of diabetes.

On the other hand, the 250 and 500 mg/kg FD treatments significantly decreased pancreatic MDA levels by 15 and 31 %, respectively, whereas the 125 mg/kg FD treatment non-significantly reduced the pancreatic MDA level in diabetic rats. Moreover, the 150 mg/kg MET treatment significantly increased the GSH content by 46 % but did not significantly affect the MDA level in the pancreas of diabetic rats (Table 7).

### Histopathological examination

**Fig. 4.**
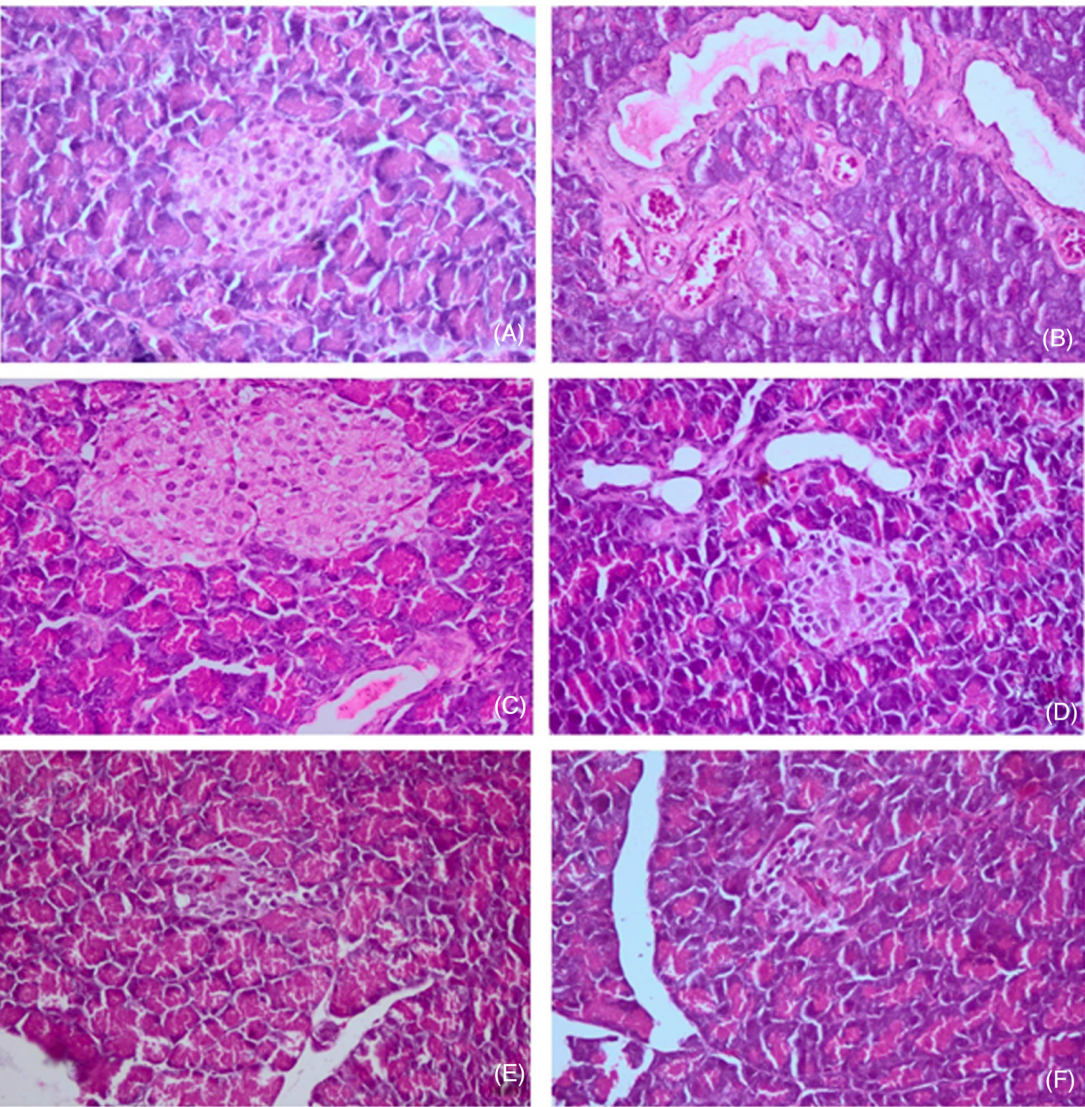
Histopathology of the pancreas of the control group (A), diabetic group (B), group treated with *Ficus deltoidei* at 125 mg/kg body weight (C), group treated with *F. deltoidei* at 250 mg/kg body weight (D), group treated with *F. deltoidei* at 500 mg/kg body weight (E) and the metformin-treated group (F).

**Fig. 5.**
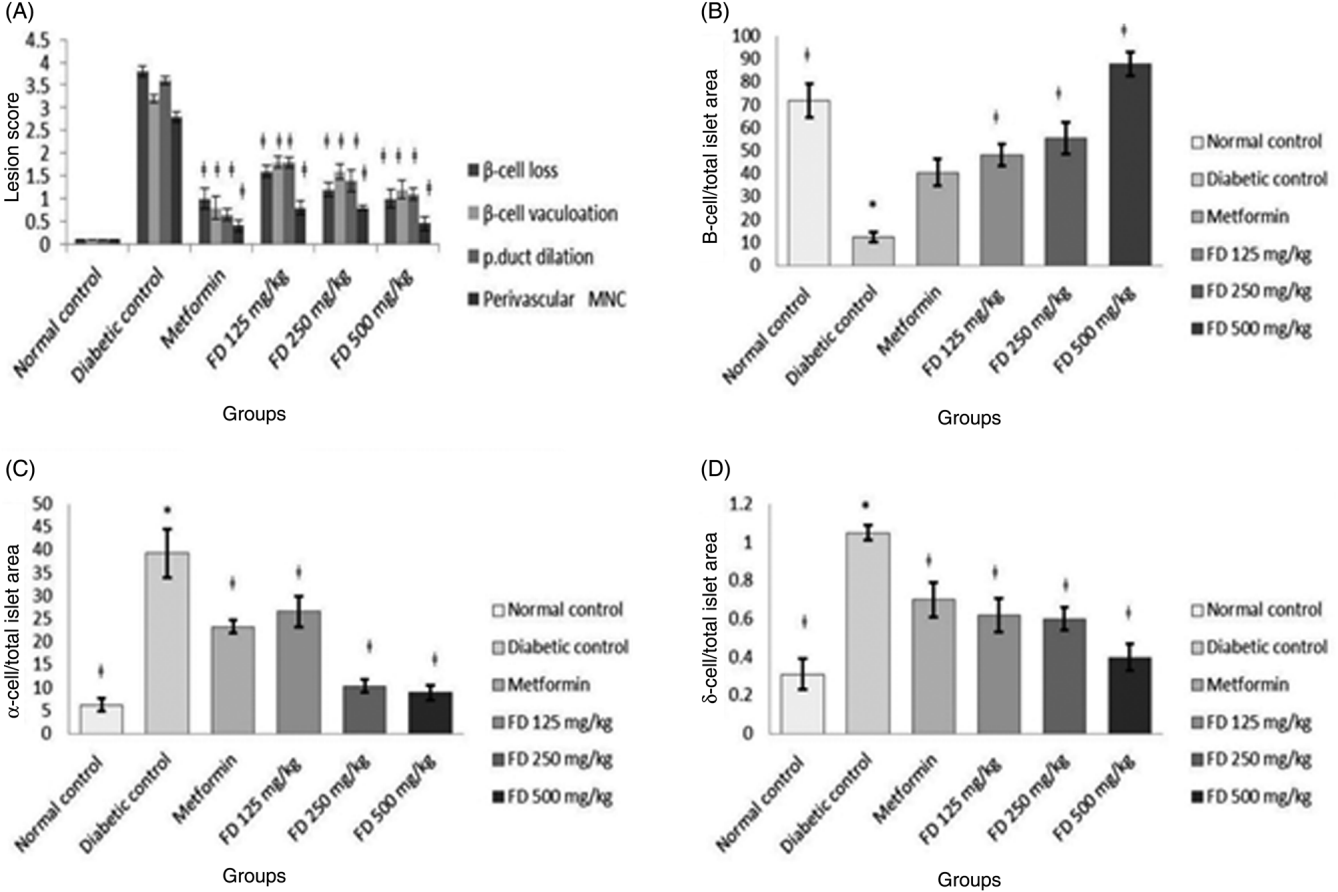
Morphometric analysis of the pancreatic islets. (A) Lesion score, β-cell/total islet area (B), α-cell/total islet area (C) and δ-cell/total islet area (D). Values are means, with standard deviations represented by vertical bars. * Mean value was statistically significantly different from that of the normal control group (*P* ≤ 0·05). † Mean value was statistically significantly different from that of the diabetic control group (*P* ≤ 0·05). FD 125 mg/kg, *Ficus deltoidei* 125 mg/kg body weight; FD 250 mg/kg, *F. deltoidei* 250 mg/kg body weight; FD 500 mg/kg, *F. deltoidei* 500 mg/kg body weight; p.duct, pancreatic duct; MNC, mononuclear cells.

Histopathological examination of the pancreas of the control group revealed a normal histology of the pancreatic acini and pancreatic islets of Langerhans (Fig. 4(A)). The islets contained a central core of β-cells surrounded in the periphery by a large mantle of α- and δ-endocrine cells. In the diabetic group, the number of β-cells in the pancreatic islets was markedly reduced (Fig. 4(B)), and the more prominent cells were α- and δ-endocrine cells. Pancreatic islets appeared disrupted. Pancreatic ducts were severely dilated, with papillary hyperplasia of the epithelial lining. Three different doses of FD markedly improved the histopathological lesions in the islet of Langerhans, particularly the β-cell loss, as shown in Figs 4(C)–(E) and 5(A). Therefore, FD had a strong effect on maintaining β-cell and insulin production in diabetic rats.

### Immunohistochemical analysis of insulin, glucagon and somatostatin protein expression and islet morphology

**Fig. 6.**
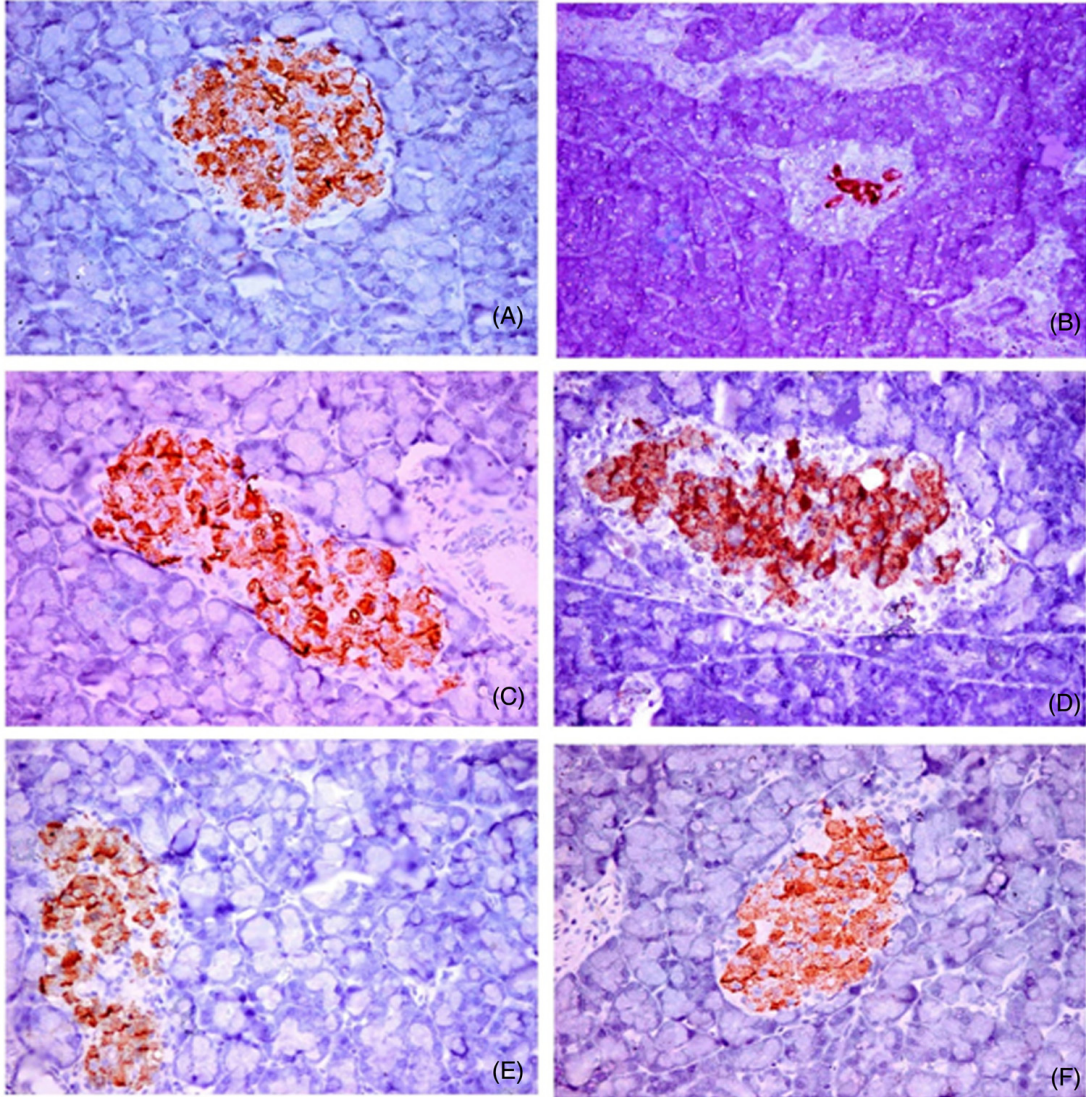
Immunohistochemical analysis of insulin protein expression and islet morphology in the control group (A), diabetic group (B), group treated with *Ficus deltoidei* at 125 mg/kg body weight (C), group treated with *F. deltoidei* at 250 mg/kg body weight (D), group treated with *F. deltoidei* at 500 mg/kg body weight (E) and the metformin-treated group (F).

**Fig. 7.**
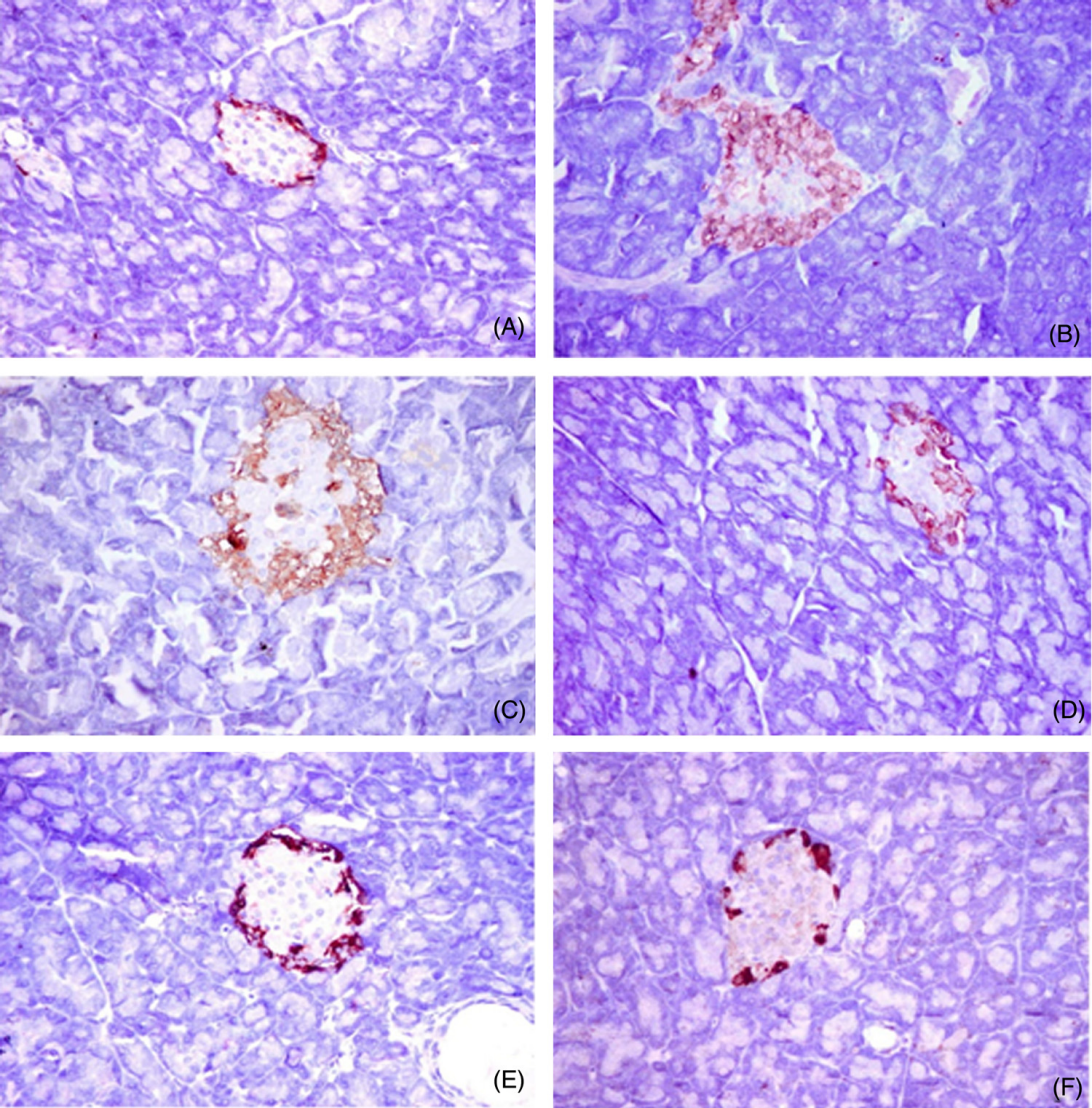
Immunohistochemical analysis of glucagon protein expression and islet morphology in the control group (A), diabetic group (B), group treated with *Ficus deltoidei* at 125 mg/kg body weight (C), group treated with *F. deltoidei* at 250 mg/kg body weight (D), group treated with *F. deltoidei* at 500 mg/kg body weight (E) and the metformin-treated group (F).

**Fig. 8.**
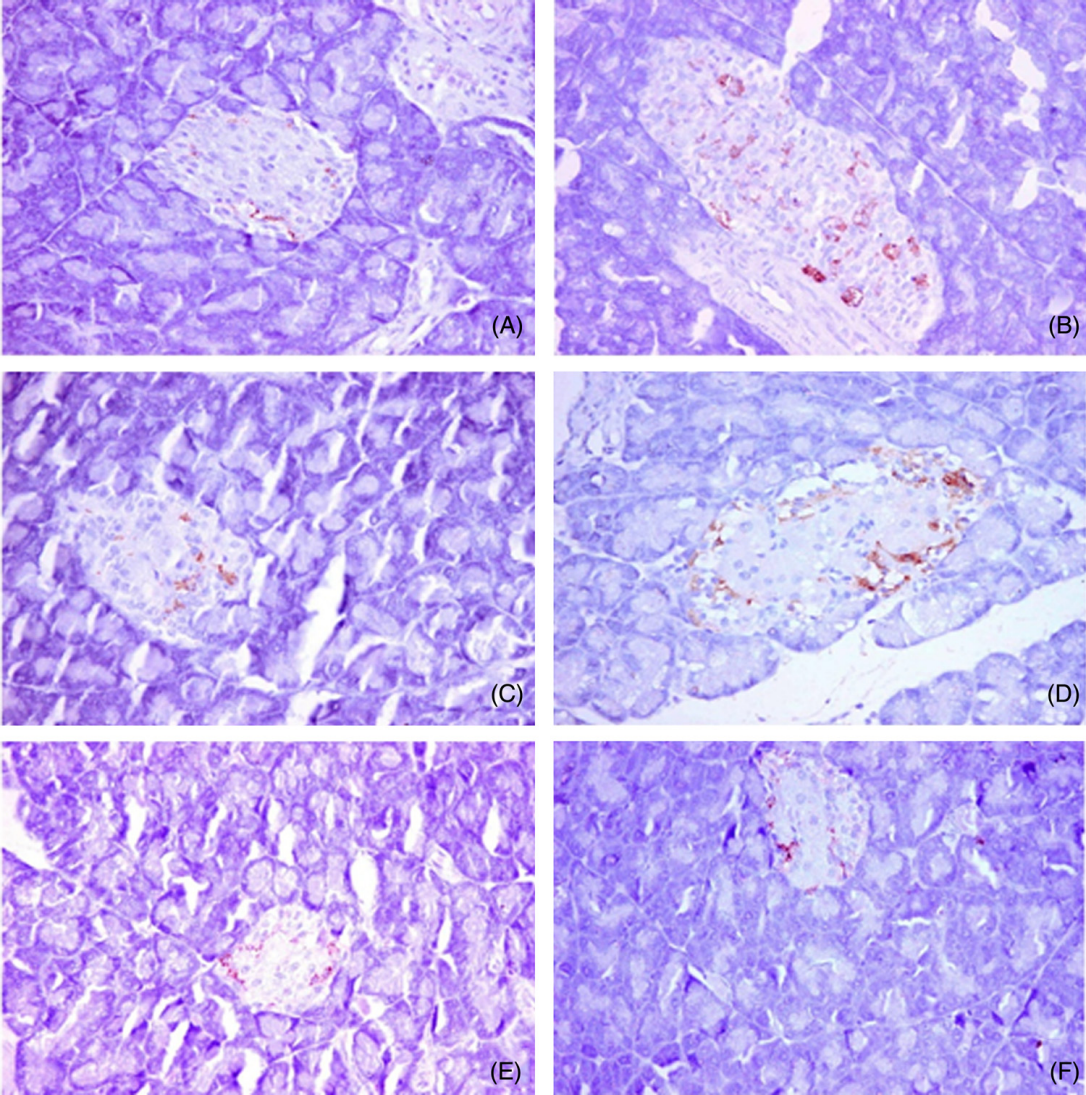
Immunohistochemical analysis of somatostatin protein expression and islet morphology in the control group (A), diabetic group (B), group treated with *Ficus deltoidei* at 125 mg/kg body weight (C), group treated with *F. deltoidei* at 250 mg/kg body weight (D), group treated with *F. deltoidei* at 500 mg/kg body weight (E) and the metformin-treated group (F).

The non-diabetic control group showed positive β-cells that occupied most of the pancreatic islets, with a diffuse distribution of the insulin content (Fig. 6(A)). Glucagon was localised in α-cells present in the peripheral area of the pancreatic islet (Fig. 7(A)). Somatostatin was localised in δ-cells that formed an incomplete ring in the pancreatic islet (Fig. 8(A)). The diabetic group exhibited a substantial decrease in the insulin content of β-cells (Fig. 6(B)), with a marked increase in the glucagon (Fig. 7(B)) and somatostatin (Fig. 8(B)) contents in the α-cells and δ-cells in the peripheral and central regions of the pancreatic islet, respectively. In the various treated groups, the insulin content (Fig. 6) of the β-cells was significantly elevated in the core of the islet, with a marked reduction in the glucagon and somatostatin contents in the α-cells and δ-cells in the islet, respectively, particularly in the core, as shown in Figs 7 and 8. The morphometric analysis of the pancreatic islets (Figs 5(B)–(D)) revealed that the percentage of insulin-positive β-cells relative to the total islet area was substantially decreased in the diabetic group compared with the non-diabetic control group. Moreover, the percentages of glucagon-positive α-cells and somatostatin-positive δ-cells relative to total islet area were significantly increased compared with the non-diabetic control group. The various treatments markedly increased the percentage of insulin-positive β-cells relative to the total islet area and markedly decreased the percentages of glucagon-positive α-cells and somatostatin-positive δ-cells relative to the total islet area (Figs 5(B)–(D)).

### Effects of *Ficus deltoidea* on glucose uptake and metabolism-related gene expression

**Fig. 9.**
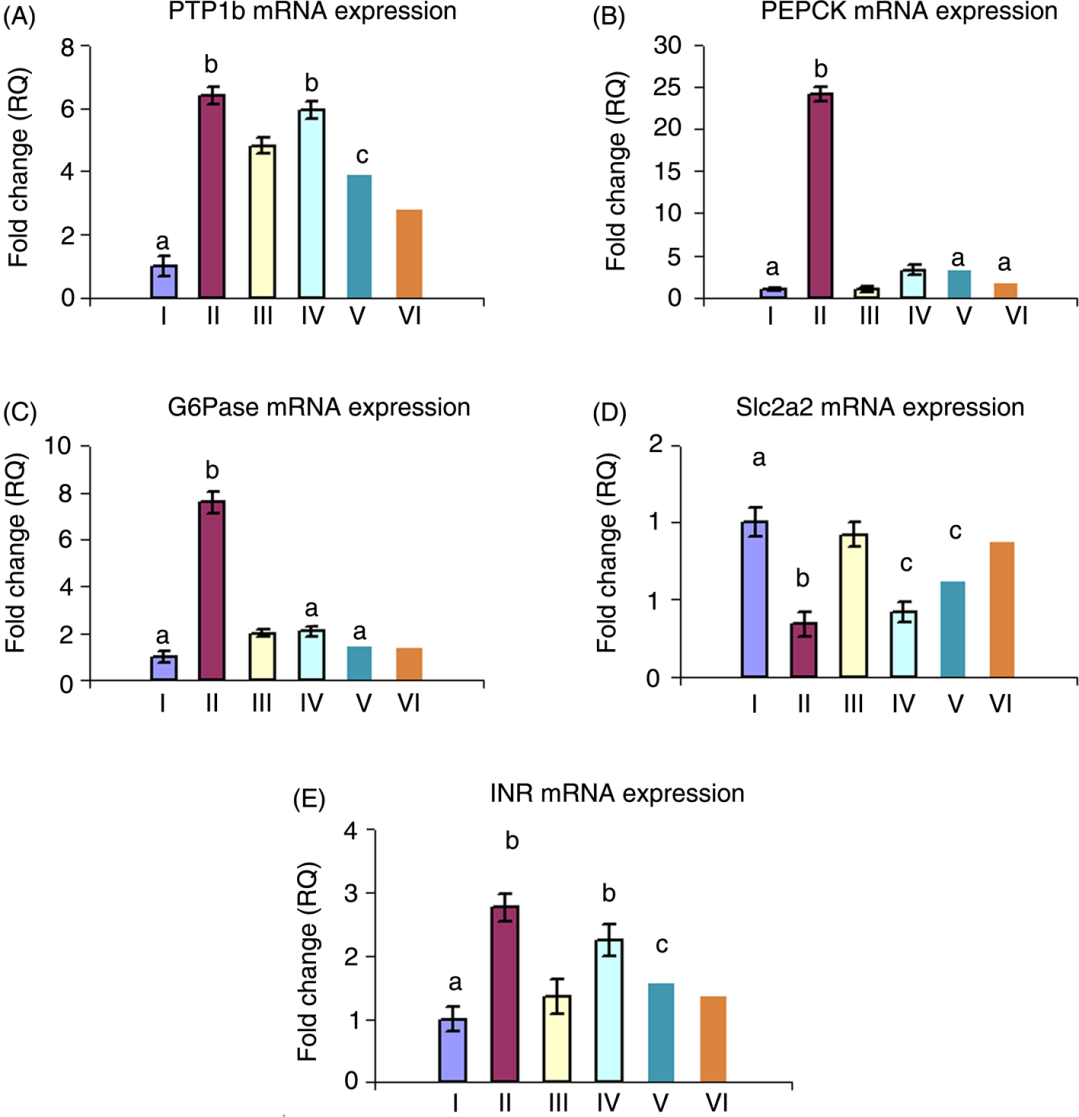
Effects of *Ficus deltoidea* on glucose uptake and metabolism-related gene expression. Relative quantification (RQ) of mRNA expression of protein tyrosine phosphatase 1B (A), phosphoenolpyruvate carboxykinase (PEPCK) (B), glucose 6-phosphatase (G6Pase) (C), GLUT2 gene (*Slc2a2*) (D) and insulin receptor (*INR*) (E). Values are means, with standard deviations represented by vertical bars. ^a,b,c^ Mean values with unlike letters were significantly different (*P* ≤0.05). Group I, control group; group II, diabetic group; group III, group treated with *F. deltoidei* at 125 mg/kg body weight; group IV, group treated with *F. deltoidei* at 250 mg/kg body weight; group V, group treated with *F. deltoidei* at 500 mg/kg body weight; group VI, metformin-treated group.

The expression of genes associated with glucose uptake and metabolism in the diabetic group was analysed (Fig. 9).

PTP1B mRNA expression levels were significantly increased in the diabetic group by 6·4-fold compared with the control group. Groups treated with MET or 125, 250 and 500 mg/kg FD exhibited a marked decrease in PTP1B expression levels to approximately 75, 92·8, 60, and 43 % of the levels in the diabetic group, respectively (Fig. 9(A)).

Expression levels of the gluconeogenic key genes, *G6Pase* and *PEPCK*, as glucose-metabolising genes, were significantly elevated in the diabetic group by 7- and 24-fold compared with the normal control level, respectively. Treatment with FD significantly and dose-dependently reduced the expression of hepatic G6Pase and PEPCK transcripts compared with the diabetic group (Figs 9(B) and (C)).

As shown in Fig. 9(D), levels of Slc2a2 mRNA were significantly decreased in the diabetic group to 34 % of the normal level. MET and 500, 250, 125 mg/kg FD treatments increased Slc2a2 expression to 92, 87, 62 and 42 % of the normal level, respectively.

The hepatic insulin receptor (INR) mRNA level was markedly increased in diabetic rats. The administration of 500 and 250 mg/kg FD normalised INR gene expression. The 125 mg/kg dose did not produce significant changes (Fig. 9(E)).

## Discussion

In the present study, we sought to reveal the role of PTP1B inhibition by FD in its antidiabetic effect at the *in vivo* and in *vitro* levels.

First, we aimed to optimise the extraction process for FD leaves and to apply bio-guided fractionation of the best extract to isolate the most active constituents. FD leaves have been previously reported to contain flavonoids, particularly luteolin and apigenin glycosides, phenolic acids such as 4-*p*-coumaroylquinic acid and terpenes such as moretenol and lupeol^(42–45)^. The 70 % ethanol extract showed the highest phenolic and triterpene contents (Table 1) and high *in vitro* PTP1B-inhibitory activity (Fig. 2). Therefore, this extract was selected to perform further purification and isolation of its major components and to investigate its antidiabetic activity *in vivo*.

Seven compounds were isolated from the 70 % ethanolic extract of FD leaves. Four of them are known compounds, which are lupeol (F1), (24E)-stigmasta-5,8-dien-3β-ol (F2), vitexin (apigenin-8C-β-d-glucoside) (F6) and isovitexin (apigenin-6C-β-d-glucoside) (F7)^(46–54)^. Two newly isolated compounds, gallic acid (F4) and chryseriol-7O-α-rhamnoside (F5), were obtained. To the best of our knowledge, this study is the first to report the isolation of compounds F4 and F5 from FD. We also report here the isolation of a novel triterpene, 3β,11β-dihydroxyolean-12-en-23-oic acid (F3), which exerted potent PTP1B inhibition (Table 2). In light of these findings, it is suggested that isolated fractions from FD are further studied so that their antidiabetic activities can be recognised.

The STZ–NA rat model of T2DM was used to investigate the antidiabetic potential effect of 70 % ethanol FD and to clarify its mechanism of action compared with MET. MET, a potent anti-diabetic drug, is a biguanide that is considered as a first-line treatment for patients with T2DM^(55,56)^.

STZ at a low dose is able to destroy parts of insulin-secreting β-cells rather than to cause complete damage. Thus, STZ is sufficient to establish T2DM^(57)^. STZ is transported into pancreatic β-cells by GLUT2 and causes DNA damage which stimulates poly (ADP-ribose) polymerase (PARP-1) enzyme to repair DNA which resulted in depletion of intracellular NAD and ATP, and subsequently cell necrosis. NA, a precursor of NAD, prevents excess STZ-induced damage of islet cells by inhibition of PARP-1 activity and increasing intracellular NAD and thus ensuring stable T2DM^(18,58)^. On this basis, a combination of STZ and NA induces light damage of pancreatic β-cells, leading to glucose intolerance. Therefore, the STZ–NA model is suggested to be closer to human T2DM and an advantageous model to evaluate the antidiabetic potential of pharmacological and natural compounds^(59)^.

In the present study, FD significantly decreased FBG and increased plasma insulin levels in diabetic rats (Table 3). These findings could be attributed to stimulation of basal and insulin-mediated glucose uptake into adipocytes and liver cells due to the insulin-mimic activity of FD^(9,11,60)^. Moreover, Farsi *et al*.^(13)^ stated that the bioactive flavone C-glycosides, vitexin and isovitexin contents of FD are efficient antioxidants, and play a crucial role in cytoprotection and scavenging of free radicals, thereby protecting the β-cells from oxidative damage, and subsequently exerting antidiabetic activity. Additionally, FD stimulates insulin secretion due to its content of water-soluble insulin-secreting compounds and its involvement in the K^+^-ATP-dependent pathway. These insulinotropic actions of FD differ according to the extract's contents of the antidiabetic compounds^(61)^.

Moreover, administration of FD (125, 250 and 500 mg/kg) to diabetic rats significantly reduced liver enzyme activities and serum total bilirubin compared with the normal control (Table 4). These findings are in agreement with previous reports^(60,62,63)^. However, FD failed to reduce the elevated serum total protein levels which may be a result of increased rate of amino acid conversion to glucose and a reduction of ribosomal protein synthesis^(64)^.

The metabolic sequelae of STZ-induced insulin insufficiency include failure of target cells to utilise glucose, increased fatty acid flux to the liver, suppressed TAG degradation, hypertriacylglycerolaemia and hypercholesterolaemia^(65–67)^, In accordance, we observed significant increases in TAG, total cholesterol and LDL levels and decreased HDL in diabetic rats. This altered lipid profile was corrected by both MET and FD (125, 250 and 500 mg/kg) (Table 5).

A substantial body of literature has confirmed the implication of oxidative stress in the pathogenesis of both types of diabetes, ranging from decreased activity of antioxidant defence mechanisms to lipid peroxidation and, ultimately, insulin resistance^(68,69)^. Therefore, antidiabetic agents with hypoglycaemic and antioxidant properties would be useful.

Remarkably, the results of the diabetic group showed a marked reduction in both the enzymic (CAT, SOD and GPx), and non-enzymatic (GSH) antioxidants with elevation of MDA level indicating the augmented STZ-induced oxidative damage in the liver and pancreas (Tables 6 and 7). These results are in line with Sheweita *et al.*^(70)^. Different doses of FD (125, 250 and 500 mg/kg) exerted significant antioxidant effects on diabetic rats, as evidenced by the increased levels of GSH and decreased levels of MDA in the liver and pancreas. Moreover, the antioxidant enzyme activities of hepatic CAT, SOD and GPx approached normal levels after treatment with FD (Table 6), whereas the pancreatic antioxidant enzyme activities increased in a dose-dependent manner (Table 7).

In the same context, histopathological and immunostaining results of pancreatic tissue confirmed the STZ-induced β-cell damage as evidenced by the disruption of islet morphology (increase in α-cells and δ-cells) and the subsequent reduction in insulin production and elevation of glucagon and somatostatin production (Figs 6 and 7)^(71,72)^. Interestingly, FD preserved β-cells from damage and maintained islet morphology. These protective effects may be due to the water-soluble insulin-secreting and α-glucosidase-inhibiting polyphenols^(9,73)^, together with other antioxidants of FD that offer protection against the early stage of diabetes^(45,74)^.

Insulin resistance is a hallmark of T2DM and obesity-associated metabolic disorders^(75)^. Insulin signal transduction is mediated by a series of molecular events, including activation of the insulin receptor through autophosphorylation of its tyrosine residues by receptor tyrosine kinase (RTK), recruitment of the downstream docking protein insulin receptor substrate 1 (IRS-1) protein, activation of phosphatidylinositol 3-kinase (PI3K) and protein kinase B (PKB; AKT), and subsequently, translocation of GLUT4 to the cell surface, leading to glucose uptake^(76,77)^. This process is negatively regulated by non-receptor protein tyrosine phosphatases (PTP) that are ubiquitously expressed in insulin-responsive tissues^(78)^. Several PTP have been implicated in modulating insulin signal transduction^(79)^. PTP1B is a negative regulator of insulin receptor and IRS-1 by hydrolysing insulin-induced tyrosine phosphorylation and subsequently induces insulin resistance^(80,81)^. Based on accumulating evidence, PTP1B inhibition in various tissues augments insulin-initiated signalling and holds great promise for the treatment of T2DM^(82–84)^.

Our study shed some light on the molecular mechanisms underlying the FD potential in amelioration of insulin resistance. First, treatment with 250 and 500 mg/kg FD significantly reduced the hepatic PTP1B mRNA overexpression in diabetic rats (Fig. 9(A)). Second, FD extracts (500 and 250 mg/kg), similar to MET, improved cellular sensitivity to insulin by normalising the hepatic insulin receptor mRNA expression that showed significant increase in the untreated diabetic rats (Fig. 9(E)). These results are consistent with previous studies^(85–87)^ showing that changes in insulin receptor expression at least partially contribute to the modulation of insulin binding in the liver of rats with STZ-induced insulin deficiency. This effect of STZ administration on hepatic insulin receptor mRNA levels was reversed by MET.

The results of both the *in vitro* PTP1B-inhibition assay and *in vivo* PTP1B mRNA expression support each other.

The *Slc2a2* gene encodes a membrane-bound, insulin-independent GLUT2, with a high glucose Michaelis constant (*K*_m_), and it is mainly expressed in the liver. Defects in the *Slc2a2* gene potentially alter glucose homeostasis^(88)^. In the present study, hepatic SLC2a2 mRNA and its protein GLUT2 were significantly decreased in the diabetic group (Figs 9(D) and 3(A)), a finding that coincides with previous studies in which STZ–NA diabetic rats exhibited a significant decrease in Slc2a2 mRNA level^(89–91)^. Down-regulation of GLUT2 observed in the present study may be attributed to the impaired insulin sensitivity and the altered glucose metabolism due to the relative insulin deficiency induced by STZ; this is in accordance with El-Abhar & Schaalan^(89)^, Al-Shaqha *et al*.^(90)^ and Rathinam & Pari^(91)^. In contrast, other studies clarified that hyperglycaemia, when associated with insulin, stimulates GLUT2^(93,94)^.

In the present study, hepatic glucose utilisation was improved in treated groups as evidenced by the up-regulation of Slc2a2 mRNA (Fig. 9(D)) and GLUT2 (Fig. 3(A)) in the liver. Consistent with the results from previous studies, Slc2a2 expression is corrected to near normal levels in groups treated with MET (Fig. 9(D)). Moreover, treatments with 500, 250 and 125 mg/kg FD dose-dependently increased the hepatic Slc2a2 expression level.

Hepatic gluconeogenesis contributes to elevations in fasting glucose levels. Based on accumulating data, gluconeogenesis rate is elevated in diabetic subjects, and its suppression provides an excellent mechanism for reducing blood glucose levels^(95,96)^. In the present study, diabetic rats showed up-regulation of mRNA expression of the rate-limiting gluconeogenic enzymes, G6Pase and PEPCK (Figs 9(B) and (C)). These results are consistent with Farsi *et al*. and Xia *et al*.^(13,94)^. As selective enhancement of insulin signalling in the liver would suppress gluconeogenesis, FD-induced improvement in insulin signalling was expected to inhibit the expression of gluconeogenesis-related genes. Notably, we observed decreased expression of G6Pase and PEPCK in the livers of both MET- and FD-treated groups, consistent with increased insulin receptor and Slc2a2 expression (Figs 9(B) and (C)). Thus, we postulate that the blood glucose-lowering effect of FD is partially attributed to the suppression of hepatic glucose output, similar to MET^(14,96)^.

### Conclusion

The present study reported two newly isolated compounds (gallic acid and chryseriol-7O-α-rhamnoside) and a novel triterpene (3β,11β-dihydroxyolean-12-en-23-oic acid) in the 70 % ethanol extract of FD with potent *in vitro* PTP1B-inhibitory activities. On the other hand, the study demonstrated for the first time that FD treatment restored insulin signalling transduction through regulation of hepatic PTP1B, and subsequently normalising insulin receptor mRNA expression. Regulation of the key gluconeogenic enzymes and Slc2a2 expression confirmed this mechanism. These findings provide theoretical evidence for FD extract to be potentially used in the management of insulin resistance in T2DM and to be a promising natural source for the development of novel antidiabetic drugs.

## Supplementary material

For supplementary material accompanying this paper visit http://dx.doi.org/10.1017/jns.2019.40. click here to view supplementary material
