# Supplementary material for: Ficus deltoidea extract down-regulates protein tyrosine phosphatase 1B expression in a rat model of type 2 diabetes mellitus: a new insight into its antidiabetic mechanism
Source: J Nutr Sci. 2020 Jan 20;9:e2. doi: 10.1017/jns.2019.40 (PMC6984126; doi:10.1017/jns.2019.40)
Supplement: Supplementary file 1 [file S2048679019000405sup001.docx]

**Table S1. Primers sequences for the target genes**

| **Gene** |  | **Primer sequence** | **Accession #** |
| --- | --- | --- | --- |
| **PTPb1** | Forward | TGGAGAAAGGCTCGTTA | NM_012637.2 |
|  | Reverse | TGCACCCTCGATCACA |  |
| **PEPCK** | Forward | GTGTCATCCGCAAGCTGAAGA | [NM_198780.3](http://www.ncbi.nlm.nih.gov/entrez/viewer.fcgi?db=nucleotide&id=61835220) |
|  | Reverse | CTTTCGATCCTGGCCACATCT |  |
| **G6Pase** | Forward | GGATCTACCTTGCGGCTCACT | [NM_013098.2](http://www.ncbi.nlm.nih.gov/entrez/viewer.fcgi?db=nucleotide&id=145207997) |
|  | Reverse | TGTAGATGCCCCGGATGTG |  |
| **Slc2a2** | Forward | TCTGTGCTGCTTGTGGAG | [XM_006232207.2](http://www.ncbi.nlm.nih.gov/entrez/viewer.fcgi?db=nucleotide&id=672042002) |
|  | Reverse | ACTGACGAAGAGGAAGATGG |  |
| ***INSR*** | Forward | TTTGTCATGGATGGAGGCTA | [XM_006248753.2](http://www.ncbi.nlm.nih.gov/entrez/viewer.fcgi?db=nucleotide&id=672071893) |
|  | Reverse | CCTCATCTTGGGGTTGAACT |  |
| **Β-actin** | Forward | ATGGTGGGTATGGGTCAG | [NM_031144.3](http://www.ncbi.nlm.nih.gov/entrez/viewer.fcgi?db=nucleotide&id=402744873) |
|  | Reverse | CAATGCCGTGTTCAATGG |  |

**Table S2. ^13^CNMR (100 MHz) of compounds F1, F2 and F3**

| **Position** | **δ_C_ ppm** | | |
| --- | --- | --- | --- |
|  | **F1** | **F2** | **F3** |
| 1 | 38.7 | 37.2 | 42.3 |
| 2 | 27.4 | 31.5 | 26.9 |
| 3 | 79.0 | 71.8 | 76.3 |
| 4 | 38.8 | 42.3 | 56.7 |
| 5 | 55.3 | 140.7 | 52.1 |
| 6 | 18.3 | 121.7 | 19.1 |
| 7 | 15.1 | 31.9 | 34.5 |
| 8 | 40.8 | 138.3 | 40.7 |
| 9 | 50.4 | 129.2 | 49.8 |
| 10 | 37.1 | 36.5 | 36.9 |
| 11 | 21.1 | 21.0 | 82.6 |
| 12 | 25.1 | 39.7 | 126.1 |
| 13 | 38.0 | 42.2 | 147.7 |
| 14 | 42.8 | 56.7 | 45.6 |
| 15 | 27.6 | 24.3 | 27.1 |
| 16 | 35.5 | 29.3 | 27.2 |
| 17 | 43.0 | 56.0 | 45.9 |
| 18 | 47.9 | 11.8 | 50.2 |
| 19 | 48.3 | 19.4 | 49.8 |
| 20 | 150.9 | 40.5 | 31.8 |
| 21 | 29.9 | 21.2 | 36.4 |
| 22 | 40.0 | 33.9 | 31.5 |
| 23 | 28.0 | 28.2 | 184.6 |
| 24 | 15.3 | 51.2 | 13.9 |
| 25 | 16.1 | 31.6 | 17.6 |
| 26 | 15.9 | 19.0 | 19.1 |
| 27 | 14.5 | 21.0 | 27.3 |
| 28 | 18.0 | 26.0 | 22.2 |
| 29 | 109.3 | 11.9 | 31.8 |
| 30 | 19.3 |  | 20.6 |

**Table S3. ^13^CNMR (100 MHz) of compounds F4, F5, F6 and F7**

| **Position** | **δ_C_ ppm** | | | |
| --- | --- | --- | --- | --- |
|  | **F4** | **F5** | **F6** | **F7** |
| 1 | 121.6 |  |  |  |
| 2 | 109.5 | 163.1 | 164.2 | 163.9 |
| 3 | 145.6 | 103.6 | 102.9 | 103.2 |
| 4 | 140.5 | 182.2 | 182.5 | 182.4 |
| 5 | 145.6 | 161.2 | 160.8 | 161.1 |
| 6 | 109.5 | 99.7 | 98.5 | 109.3 |
| 7 | 168.3 | 164.4 | 163.5 | 163.7 |
| 8 |  | 95.2 | 105.0 | 94.0 |
| 9 |  | 157.1 | 156.4 | 156.6 |
| 10 |  | 105.5 | 104.5 | 103.8 |
| 1` |  | 121.6 | 122.0 | 121.5 |
| 2` |  | 110.6 | 129.4 | 128.9 |
| 3` |  | 153.0 | 116.5 | 116.4 |
| 4` |  | 148.5 | 161.5 | 161.6 |
| 5` |  | 116.0 | 116.5 | 116.4 |
| 6` |  | 120.7 | 129.4 | 128.9 |
| 3`-OCH_3_ |  | 56.2 |  |  |
| 1`` |  | 100.1 | 73.8 | 73.5 |
| 2`` |  | 72.1 | 71.3 | 71.0 |
| 3`` |  | 72.4 | 79.1 | 79.4 |
| 4`` |  | 74.1 | 71.0 | 70.6 |
| 5`` |  | 69.8 | 82.2 | 82.0 |
| 6`` |  | 17.9 | 61.7 | 61.9 |

**Table S4. Characterization of Bioactive compounds isolated from FD**

| **Compound F1** | White microcrystalline powder (75 mg). m.p. (210-212°C). R*_f_* values (0.64 in S2); it gives purple colour with PA spray reagent.  ^1^HNMR (400MHz CDCl_3_): revealed signals for seven tertiary methyl groups at δ 0.78 (3H, s, Me-28), 0.81 (3H, s, Me-23), 0.85 (3H, s, Me-24), 0.92 (3H, s, Me-25), 0.96 (3H, s, Me-27), 1.05 (3H, s, Me-26) and 1.70 (3H, s, Me-30), 3.21 (1H, dd, J=3Hz, 6 Hz indicative of H-3 is α- oriented) and 4.58 (1H, s, H-29b), 4.70 (1H, s, H-29a). |
| --- | --- |
| **Compound F2** | White needle crystals (70 mg), m.p. (169-170°C). R*_f_* values (0.52 in S2). It gives violet colour with PA spray reagent.  ^1^H-NMR: δ (300 MHz, CDCl_3_) 0.70 (3H, d, *J*=5.4 Hz, Me-21), 0.83 (3H, t, *J*=6.3, Me-29), 0.90 (3H, d, *J*=6.4 Hz, Me-26), 0.93 (3H, d, *J*=6.3 Hz, Me-27), 1.03 (3H, s, Me-18), 1.28 (3H, s, Me-19), 3.50 (1H, m, H-3) and 5.37 (1H, br.s., H-6) ppm. |
| **Compound F3** | White needle crystals (110 mg), m.p.308-309°C, R*_f_* values (0.46 in S2); it gives purple colour with PA spray reagent**.**  ^1^H-NMR: δ (400 MHz, DMSO) 0.68 (3H, s, Me-28), 0.72 (3H, s, Me-29), 0.85 (3H, s, Me-30), 0.88 (3H, s, Me-27), 0.90 (3H, s, Me-26), 1.06 (3H, s, Me-25), 1.09 (3H, s, Me-24), 2.72 (1H, m, H-18), 3.17 (1H, dd,*J*=12.0,4.8HZ, H-3), 4.28 (1H, dd,*J*=9.6,3.2 Hz, H-11) and 5.16 (1H, d, *J*=3.2 Hz, H-12) ppm. |
| **Compound F4** | White powder (130 mg), R*_f_* 0.59 in S3. It gives greenish yellow color with NP-PEG spray reagent. ^1^H-NMR δ ppm (400 MHz, DMSO) 7.10 (2H, s, H-2,6). |
| **Compound F5** | Yellow microcrystalline powder (43 mg). It gives yellow color with NP-PEG spray reagent. R*_f_* 0.51 in S3. ^1^H-NMR δ ppm (400 MHz, DMSO). 1.1 (3H,d,*J=*6.6, CH_3_-6``), 3.72 (3H,s, OCH_3_-3`), 5.25 (1H,d, *J*=1.2 Hz, H-1``), 6.28 (1H,s, H-6), 6.53 (1H,s,H-8), 6.73 (1H,d,*J*=8.4, H-5`), 6.84 (1H,dd,*J*=1.2, 8.4, H-6`), 7.42 (1H, d, *J*=1.2, H-2`). |
| **Compound F6** | Yellow microcrystalline powder (62 mg). It gives yellow color with NP-PEG spray reagent. R*_f_* 0.49 in S3. ^1^H-NMR δ ppm (400 MHz, DMSO). 4.68 (1H,d, J=9.8 Hz, H-1``), 6.40 (1H,d, *J*=1.8, H-6), 6.70 (1H,d, *J*=1.8, H-8), 6.78 (1H,s,H-3), 6.88 (2H,d,*J*=8.4, H-2`,6`), 8.02 (2H, d, *J*=8.4, H-3`,5`). |
| **Compound F7** | Yellow microcrystalline powder (160 mg). It gives yellow color with NP-PEG spray reagent. R*_f_* values (0.42 in S3). ^1^H-NMR δ ppm (400 MHz, DMSO) 4.58 (1H,d, J=9.8 Hz, H-1``), 6.52 (1H,s, H-8), 6.79 (1H,s,H-3), 6.92 (2H,d,*J*=8.8, H-2`,6`), 7.92 (2H, d, *J*=8.8, H-3`,5`). |
